# On-demand, hospital-based, severe acute respiratory coronavirus virus 2 (SARS-CoV-2) genomic epidemiology to support nosocomial outbreak investigations: A prospective molecular epidemiology study

**DOI:** 10.1017/ash.2023.119

**Published:** 2023-03-08

**Authors:** Patrick Benoit, Gisèle Jolicoeur, Floriane Point, Chantal Soucy, Karine Normand, Philippe Morency-Potvin, Simon Gagnon, Daniel E. Kaufmann, Cécile Tremblay, François Coutlée, P. Richard Harrigan, Isabelle Hardy, Martin Smith, Patrice Savard, Simon Grandjean Lapierre

**Affiliations:** 1 Department of Microbiology, Infectious Diseases and Immunology, Université de Montréal, Montréal, Québec, Canada; 2 Department of Medicine, Université de Montréal, Montréal, Québec, Canada; 3 Immunopathology Axis, Centre de Recherche du Centre Hospitalier de l’Université de Montréal, Montréal, Québec, Canada; 4 Infection Prevention and Control Service, Centre Hospitalier de l’Université de Montréal, Montréal, Québec, Canada; 5 Infectious Diseases Service, Centre Hospitalier de l’Université de Montréal, Saint-Denis, Montréal, Québec, Canada; 6 Molecular Biology Service, Centre Hospitalier de l’Université de Montréal, Saint-Denis, Montréal, Québec, Canada; 7 Department of Medicine, University of British Columbia, Vancouver, British Columbia, Canada; 8 Department of Biochemistry and Molecular Medicine, Université de Montréal, Montréal, Québec, Canada

## Abstract

**Objectives:**

We evaluated the added value of infection control-guided, on demand, and locally performed severe acute respiratory coronavirus virus 2 (SARS-CoV-2) genomic sequencing to support outbreak investigation and control in acute-care settings.

**Design and setting:**

This 18-month prospective molecular epidemiology study was conducted at a tertiary-care hospital in Montreal, Canada. When nosocomial transmission was suspected by local infection control, viral genomic sequencing was performed locally for all putative outbreak cases. Molecular and conventional epidemiology data were correlated on a just-in-time basis to improve understanding of coronavirus disease 2019 (COVID-19) transmission and reinforce or adapt control measures.

**Results:**

Between April 2020 and October 2021, 6 outbreaks including 59 nosocomial infections (per the epidemiological definition) were investigated. Genomic data supported 7 distinct transmission clusters involving 6 patients and 26 healthcare workers. We identified multiple distinct modes of transmission, which led to reinforcement and adaptation of infection control measures. Molecular epidemiology data also refuted (n = 14) suspected transmission events in favor of community acquired but institutionally clustered cases.

**Conclusion:**

SARS-CoV-2 genomic sequencing can refute or strengthen transmission hypotheses from conventional nosocomial epidemiological investigations, and guide implementation of setting-specific control strategies. Our study represents a template for prospective, on site, outbreak-focused SARS-CoV-2 sequencing. This approach may become increasingly relevant in a COVID-19 endemic state where systematic sequencing within centralized surveillance programs is not available.

**Trial registration:**

clinicaltrials.gov identifier: NCT05411562

Acute-care and long-term care facility–associated severe acute respiratory coronavirus virus 2 (SARS-CoV-2) infections were reported to be as high as 44% of cases in the early days of the pandemic, and studies have demonstrated the increased morbidity and mortality in vulnerable hospitalized patient populations.^1–3^ Nosocomial outbreaks hinder the pandemic response and limit the ability of medical institutions to respond to other population needs. Despite effective infection prevention and control (IPAC) programs, limiting nosocomial transmission proves challenging because patients and healthcare workers (HCWs) are constantly at the interface of healthcare setting and community transmission clusters.^4–6^ Coronavirus disease 2019 (COVID-19) can be hospital acquired through multiple mechanisms such as transmission from an infected HCW to patients, between HCWs, from infected visitors to HCWs or patients, or between patients when they share a common environment.^7,8^ In a pandemic context, with high community viral transmission, infection clusters within healthcare institutions frequently represent aggregated community-acquired infections.^6^ This situation highlights the potential value of rapidly accessible complementary molecular epidemiology data to refine understanding of transmission and to help delineate true nosocomial events from contemporary community acquisitions. In this study, we performed SARS-CoV-2 genomic sequencing locally and on demand to provide just-in-time information complementing IPAC investigations of suspected COVID-19 nosocomial outbreaks.

## Methods

### Study setting, infection control measures, and SARS-CoV-2 testing

This study was conducted in the Centre Hospitalier de l’Université de Montréal (CHUM) between April 2020 and October 2021. All conventional epidemiology investigation and viral sequencing were performed, and acted upon, prospectively as these outbreaks were unfolding and were clinically suspected. Located in Montreal, CHUM is a tertiary-care reference hospital with 770 individual rooms and ∼14,000 employees.

Best-practice infection control measures were implemented throughout the study.^9^ All 32,476 patients admitted during the study period were systematically tested upon hospitalization and were retested if new-onset COVID-19–compatible symptoms appeared. COVID-19 patients were isolated in individual rooms on cohort wards. HCWs were required to self-monitor symptoms and had access to same-day testing, after which they were removed from the workplace while awaiting testing results. If positive, a 10–14-day quarantine and symptom recovery period was implemented throughout the study (initially 14 then 10 days off work), and this period was extended to a minimum of 21 days for those who were immunocompromised, given the possibility of prolonged viral shedding.^10^ A negative-control polymerase chain reaction (PCR) test was initially required to reintegrate the work place, but this recommendation was dropped during the study given the emerging evidence suggesting the limited added value of this approach.^11^ Those in close contact (ie, <2 m for >10 minutes without mask) with contagious HCWs were clinically assessed and were required to quarantine for 14 days if not fully vaccinated. Other basic practices in infection control were also enhanced and systematically implemented: handwashing, wearing procedure masks and face shields when interacting with hospitalized patients or other HCWs, wearing specific PPE while caring for COVID-19 patients, enhanced cleaning and disinfection routines, visitor restrictions, social distancing in common areas for HCWs, and restrictions regarding in-person gatherings and meetings for HCWs. All testing was performed using RT-PCR on oral and nasopharyngeal swabs samples (cobas SARS-CoV-2 Test, Roche Diagnostics, Laval, Canada).^12^

### Surveillance and outbreak investigation

**Fig. 1.**
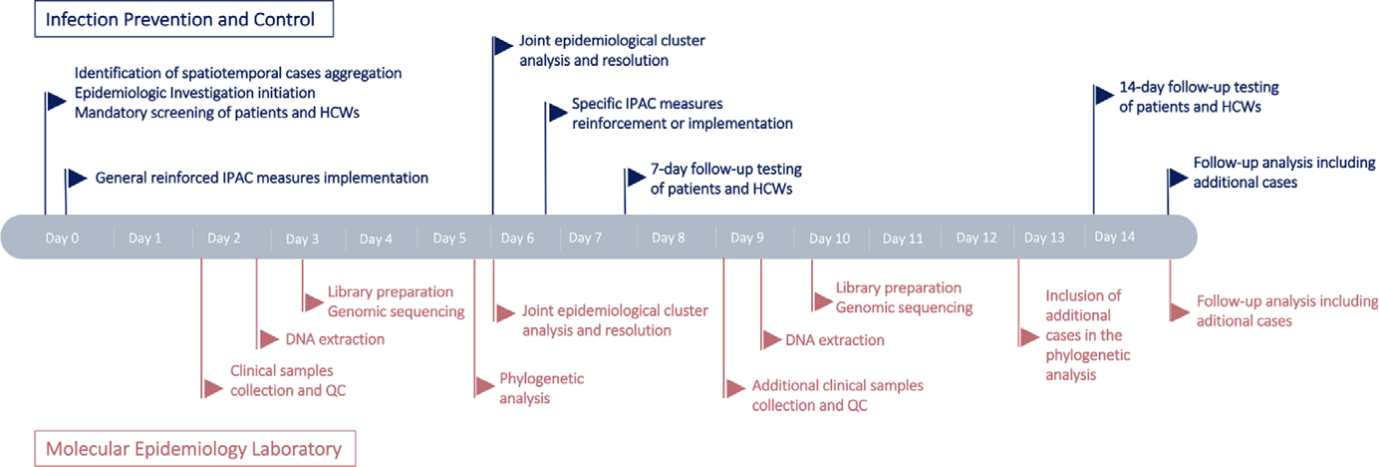
SARS-CoV-2 nosocomial outbreak epidemiological and molecular investigation. COVID-19 nosocomial outbreak investigation workflow highlighting significant steps of the collaborative work between IPAC service (blue) and the local molecular epidemiology laboratory (red). Following each wave of genomic sequencing, face-to-face meetings were held to share updates on IPAC service transmission hypotheses and molecular-laboratory-generated phylogenetic analyses. After collaborative resolution of putative outbreaks in the cases of refuted or further supported transmission, respectively, additional investigation efforts were discontinued or necessary corrective measures were implemented. Note. HCW, healthcare worker; QC, quality control; IPAC, infection prevention and control.

All positive tests among patients and HCWs were identified from the hospital laboratory information system and were reviewed by the IPAC service and hospital health safety office. A possible outbreak was defined as 2 or more infected individuals (HCWs and/or patients) sharing a spatiotemporal link within the institution. The contagious period used to establish those links was defined as 48 hours before to 10 days after a positive test or symptom onset. When outbreaks were suspected on a ward or service, all patients and HCWs were clinically monitored and systematically tested on days 0, 7, and 14 and at symptom onset if applicable. All positive HCWs completed a name-generating questionnaire to identify potential close contacts without effective PPE in the 14 days prior to their positive test or symptom onset. Hospital admission records and HCW work schedules were reviewed to identify potential patient–HCW contacts. Based on clinical and COVID-19 testing information, line lists were generated for every outbreak; initial transmission hypotheses were formulated; the described snowball sampling strategy was implemented; and positive samples were locally sequenced in real time (Fig. 1).

### SARS-CoV-2 genomic sequencing and molecular epidemiology analyses

All available RT-PCR positive samples from included patients and HCWs were retrieved and processed for viral sequencing on MinION sequencing technology (Oxford Nanopore Technologies, Oxford, UK), and genomic analyses were performed using the ARTIC V3 pipeline (see Supplementary material 1).^13,14^ We defined “likely” or “possibly” related cases based on viral genomic relatedness. We used genomic “mutations” or single-nucleotide polymorphism (SNP) thresholds from retrospective studies that included genomic characterization of outbreak isolates. Viral genomes 1 and 2 SNPs apart per 2-week period between clinical sampling were respectively considered as likely or possibly related, respectively (Supplementary Material 2).^15–17^ Our study preceded the emergence of the SARS-CoV-2 δ (delta) variant, for the most part, and entirely predated the emergence of the SARS-CoV-2 ο (omicron) variant and subvariants. No such variants were found in the putative outbreak and control isolates. All our study isolates were clades 20-A, 20-B, and 20-C. When regrouping putative transmission isolates based on SNP thresholds, all regrouped isolates also belonged to the same clade as expected.

This study protocol was made publicly available on ClinicalTrials.gov under ID NCT05411562. The study results are reported according to the Strengthening the Reporting of Molecular Epidemiology for Infectious Diseases (STROME-ID) guidelines.^18^ SARS-CoV-2 sequences from this study are available in GenBank under continuous accession numbers OM540759 to OM540803.

This study was performed using clinical information and samples provided to our IPAC service and clinical microbiology laboratory as part of routine outbreak investigations. The study did not require individual informed consent but was approved by the CHUM Ethic Committee (no. 2021-9253, 20.270).

## Results

### Outbreak investigations

**Fig. 2.**
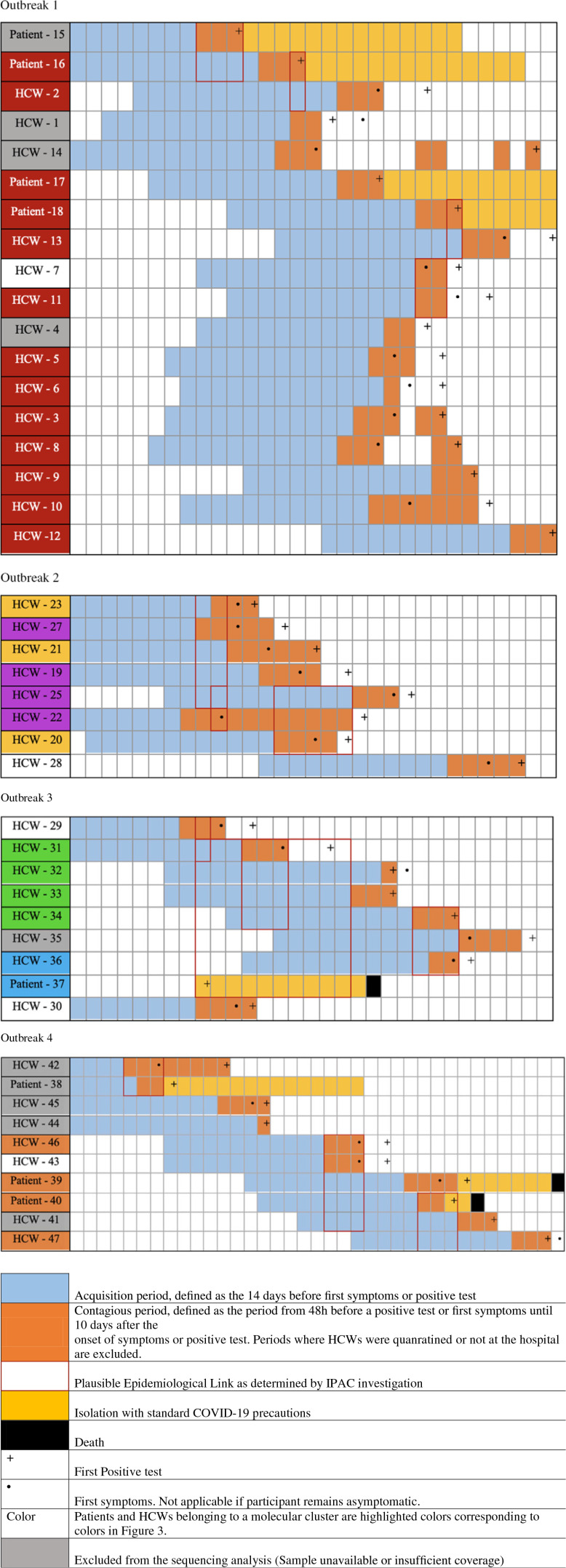
Outbreak case distribution including clinical, epidemiological, and molecular data. Case distribution of 4 selected outbreaks representing different transmission patterns (ie, between HCWs, from HCWs to patients, and from patients to HCWs). Patients and HCWs belonging to a molecular cluster are highlighted in colors matching colors used in Fig. 3. Note. HCW, healthcare worker; IPAC, infection prevention and control.

**Fig. 3.**
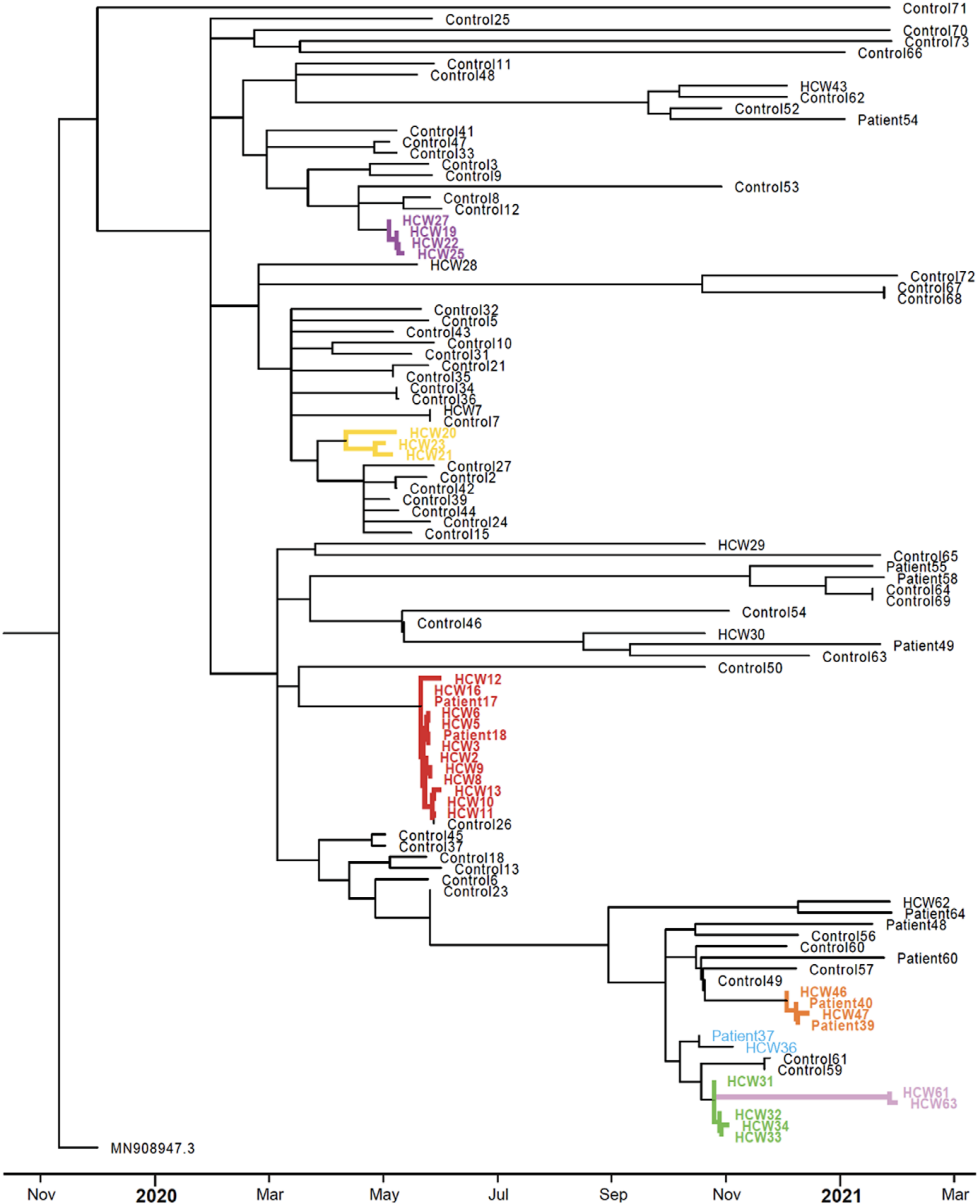
Phylogenetic analysis of SARS-CoV-2 isolates associated with nosocomial outbreaks. Phylogenetic tree showing the genetic relationship or distances between the 45 SARS-CoV-2 isolates included in the 6 putative outbreaks and randomly selected contemporary hospital and community isolates. Maximum likelihood tree generated with TreeTime software (version 0.9.2).^28^ Patients and healthcare workers (HCW) belonging to a molecular cluster are highlighted in colors matching colors used in Figure 2.

Between April 1, 2020, and October 31, 2021, ∼1,150 cases of COVID-19 were hospitalized in the study institution. Overall, 6 distinct putative outbreaks involving 59 infected individuals (19 patients and 40 HCWs) were investigated. Unfortunately, 7 positive samples could not be retrieved either because the COVID-19 diagnosis had been confirmed in a distinct institution prior to admission or because the sample had been referred to a public health surveillance laboratory before local outbreak investigation. In total, 52 samples were available, and a high-quality viral genomic sequence was generated for 45 (87%). Molecular investigations supported transmission in 32 cases (71.1%) and refuted transmission in 13 cases (28.9%). Person-to-person transmission was supported by molecular investigations in all but 1 of the epidemiologically suspected outbreaks. Multiple theoretical routes of infection did coexist and included transmission from HCWs to patients (outbreaks 1 and 4), from patient to HCW (outbreak 3), and between HCWs (outbreaks 1, 2, 3, 4, and 6). Even with extensive snowball testing for secondary case finding, no patient-to-patient transmission was confirmed. Summary information, case distribution charts, and phylogenetic analyses for all investigated outbreaks are presented in Table 1 and Figures 2 and 3.

**Table 1.** Outbreaks Characteristics and Investigation Outcomes^a^

	Type of Unit	Patients, No.	IPAC Transmission Hypotheses	Successfully Sequenced Genomes, No.	Insufficient Coverage Samples, No.	Confirmed Transmission Cluster, No.	Cluster Patients, No.	Molecular Transmission Conclusion	Corrective Measures Implemented
		HCWs, No.			Unavailable Samples, No.		Cluster HCWs, No.		
1	Geriatric unit Cold unit	4	Between HCWs Between patients	14	4	1	3	From HCW to patient Between HCWs	Prevention measures between HCWs Hospital hygiene measures on COVID-19 units
		14			0		10		
2	Acute COVID	0	Between HCWs	8	0	2	0	Between HCWs	Prevention measures between HCWs
		8			0		7		
3	Acute COVID	1	From patients to HCWs	8	0	2	1	From patient to HCW Between HCWs	Review of infection prevention measures and PPE for infected patients on HFNC
		8			1		5		
4	Acute-care Cold unit	3	From an HCW to a patient	5	3	1	2	From HCW to patient Between HCWs	Prevention measures between HCW and patients
		7			2		2		
5	In- and outpatient hemodialysis Cold unit	5	Between patients	4	0	0	0	No transmission	None necessary
		0			1		0		
6	Outpatient hemodialysis Cold unit	6	Between patients Between patients and HCWs	6	0	1	0	Between HCWs	Prevention measures between HCWs
		3			3		2		

a Summary of the outbreaks investigations outcomes. COVID-19 patients were placed in cohorts on dedicated wards (COVID-19 units). A cold unit refers to an hospitalization ward where patients are confirmed to be COVID-19 negative upon admission. Successfully sequenced samples represent minimum of 85% or 50% genomic coverage to respectively suggest or refute transmission.

### Impact on infection control

Molecular testing led to several changes in policy. Documenting HCW infections in eating areas supported specific recommendations for increased distancing during personnel breaks: delocalizing eating areas within work units, establishing a maximum number of workers taking breaks in the same room at the same time, and avoiding significant break schedules overlaps. Patient-to-HCW transmission associated with high-flow nasal cannula (HFNC) challenged the efficacy of standard PPE precautions for those patients and led to specific recommendations for hospitalization of patients on intensive oxygen support within negative-pressure rooms at all times. Confirming that COVID-19 transmission from HCWs to patients hospitalized for longer than the viral incubation period raised significant attention from the medical personnel. This finding led to specific communications to practitioners, including the recommendation for systematic COVID-19 retesting as part of fever or septic work-ups in patients recently hospitalized.

### Turnaround time and costs

Outbreak investigations were generally carried out over 1 week and were completed after 14 days when all follow-up systematic screening samples had been tested (Fig. 1). Epidemiological investigations conducted by the IPAC team and the hospital health safety team were reported to the molecular epidemiology laboratory within 2 days. Collection of outbreak and control samples with sufficient viral loads was performed on the same day. Genomic analyses validation (SNP distance matrix and phylogenetic trees building) took between 6 and 8 hours, depending on the number of cases involved in the outbreak. Most often, phylogenetic data were communicated to the IPAC team the day after the analysis, and appropriate and corrective measures were implemented immediately. If additional cases were suspected and identified, this process was iteratively repeated, adding newly sequenced genomes to previous bioinformatics analyses. Within this workflow, the turnaround time from positive COVID-19 test to sequencing was 3 days (minimum) for positive patients during the outbreak investigation. For patients who initially triggered the investigation, turnaround times could be longer and included the time necessary for spatiotemporally aggregated cases to be diagnosed and flagged by IPAC teams (Fig. 2).

Although this study was not designed to provide cost-effectiveness metrics, locally performed molecular testing and DNA sequence analysis represented a net cost of $146 CAD (US$109.47) per COVID-19 case, which included human resources and consumables.

## Discussion

We demonstrated the feasibility and value added of prospective, on-demand and hospital-based viral genomic sequencing to support nosocomial COVID-19 outbreak investigations. We established a rapid workflow from initial clinical suspicion of nosocomial transmission to sequencing and result feedback to IPAC teams. This “ask questions first, sequence later” approach allowed us to accelerate sequencing turnaround time for epidemiologically relevant samples and avoid unnecessary spending on sequencing of community-acquired infections. Viral genomic data either strengthened or refuted clinical suspicions of nosocomial transmission, which was otherwise unachievable when relying strictly on clinical investigations. By combining SARS-CoV-2 genomic relatedness with symptom temporality and positive testing dates, transmission direction was inferred with increased certainty.

For some outbreaks, understanding local transmission dynamics led to the implementation of specific control measures. Initial transmission between employees in eating areas led to secondary transmission to hospitalized patients in outbreak 1. Additional social distancing in break and eating areas was implemented, and the recommendation of avoiding presentism was reinforced following this outbreak investigation. A COVID-19–positive patient receiving HFNC oxygen support in a single patient room was initially suspected of being part of an oubreak, which led to the regrouping of 8 HCWs. Molecular testing confirmed transmission from this patient to 1 HCW and revealed that 4 other HCWs were forming a distinct transmission cluster (outbreak 3). Although this was an isolated case, IPAC teams cautiously recommended that COVID-19 patients requiring HFNC be isolated in negative-pressure rooms. Nosocomial infections were confirmed on multiple hospitalization wards (outbreaks 1, 3, and 4). In addition to reinforcing standard measures for HCWs, masking of patients (double masking) was implemented and systematic COVID-19 retesting as part of fever or septic work-ups in patients hospitalized for >14 days was recommended to clinicians.

Our data indicate that the highest risk of infection for HCWs was related to interactions among colleagues. Most genetically supported transmission events occurred between HCWs, whereas potential HCW infection originating from a patient was suspected only once. For example, in the only investigated outpatient setting, the hemodialysis clinic, the molecular investigation supported transmission between HCWs, but SARS-CoV-2 viruses detected in patients were genetically unrelated and hence were suspected of being community acquired in outbreak 6.

In other outbreaks, real-time molecular epidemiology data simply validated the work of IPAC teams, improved their understanding of transmission dynamics, and supported their efforts to reinforce preventive measures. In two investigations, genomic data allowed the dissociation of cases between several coexisting outbreaks and outbreaks with a single transmission chain (outbreaks 2 and 3). In the investigation of outbreak 5, molecular testing refuted all potential transmission links. In 29% of all cases, spatiotemporally clustered cases were genetically unrelated, which ended the outbreak investigations more rapidly. Restricting the size of outbreaks to truly related cases allowed IPAC teams to better allocate resources and focus efforts on additional testing of true nosocomial transmission clusters. Not having to pursue additional patient and HCW testing, as well as limiting repeated audits and training on affected wards, was beneficial because IPAC resources were limited in this pandemic-response context.

Few studies have used SARS-CoV-2 sequencing to prospectively identify viral clusters in nosocomial outbreaks and to help hypothesize on transmission mechanisms.^19–21^ Turnaround times of sequencing analyses and specific impacts on outbreak control strategies were not reported.

In most healthcare facility-based studies to date, viral sequencing has been used retrospectively to map viral transmission.^22–25^ Although highly informative, such an approach cannot inform clinicians how to tailor infection control measures in real time. Meredith et al^26^ previously reported on SARS-CoV-2 genomic surveillance in a United Kingdom hospital with rapid turnaround times and weekly feedback to IPAC teams.^27^ In this study, sequencing was delocalized in a surveillance approach irrespective of outbreak suspicion, and molecular cluster analyses were carried out without a priori knowledge of ongoing transmission. This procedure led to as much as 22% of identical SARS-CoV-2 genomes having no epidemiological link. They report that fluid communication between infection control and laboratory teams as well as turnaround time to ensure provision of actionable information are important challenges. In our study, performing end-to-end genomic sequencing and analyses on site rather than outsourcing this process to centralized reference laboratories improved flexibility and turnaround times, which are critical if viral sequencing is expected to improve outbreak management. This strategy fostered continuous interaction between the IPAC and molecular epidemiology teams, which increased the fluidity of our sequencing workflow and outbreak management. Limiting local and rapid sequencing analyses to outbreak suspicions also represents a more cost-effective strategy specifically now that many countries have entered a COVID-19 endemic state that is expected to affect the attention and resources dedicated to this disease within surveillance programs and laboratories.

This study had several limitations inherent to prospective outbreak management within routine clinical care. Epidemiological investigations were contingent on participants not forgetting or concealing important information to IPAC and health and safety office. Some samples could not be retrieved for genomic sequencing because too little material remained following PCR testing or sample referral between institutions. For low-viral-load clinical samples, insufficient sequencing coverage limited the ability to support transmission. In our study, 7 (13%) of 52 available samples were excluded because the coverage was below our threshold. Because we performed sequencing as needed when an outbreak was suspected and not systematically for all positive cases in healthcare workers or patients, it is possible that we neglected unknown epidemiological links or outbreaks, as has been noted in other studies.^20,26^ Transmission involving visitors could not be confirmed because those were not captured by our inclusion strategy. Previous studies have suggested that assessing within host viral diversity can enhance the resolution of our understanding of SARS-CoV-2 transmission dynamics by confirming contemporary existence of minority viral variants in epidemiologically clustered cases. In our study, no specific approach was used to assess the possibility of mixed infections and their impact on phylogenetic analyses. However, given the detailed clinical and PCR testing history available for all included cases, we doubt that this scenario could have occurred at a high enough rate within our population to affect our conclusions. Lastly, even though sequencing analysis is increasingly available and democratized, our study has limited external validity for centers without molecular epidemiology expertise or resources to perform such analyses.

Traditional and molecular epidemiology approaches to SARS-CoV-2 transmission mapping are synergistic as only with their combination can outbreak dynamics and transmission direction are fully understood. Local, on-demand, and prospective viral genomic sequencing to support investigation of COVID-19 nosocomial outbreaks is both feasible and beneficial to refute or validate transmission hypotheses and to tailor infection prevention and control measures. The proposed approach yields actionable molecular information and fosters interactions between clinical and laboratory teams in order to resolve outbreaks in a critical period.

## Supplementary material

For supplementary material accompanying this paper visit http://doi.org/10.1017/ash.2023.119. click here to view supplementary material
