# Supplementary material for: On-demand, hospital-based, severe acute respiratory coronavirus virus 2 (SARS-CoV-2) genomic epidemiology to support nosocomial outbreak investigations: A prospective molecular epidemiology study
Source: Antimicrob Steward Healthc Epidemiol. 2023 Mar 8;3(1):e45. doi: 10.1017/ash.2023.119 (PMC10028942; doi:10.1017/ash.2023.119)
Supplement: Supplementary file 1 [file S2732494X23001195sup001.docx]

**On-Demand Point-of-Care SARS-CoV-2 Genomic Sequencing to Support Nosocomial Outbreak Investigations: A Prospective Molecular Epidemiology Study in Montreal**

Table of Contents

[Supplementary Material 1 – SARS-CoV-2 genomic sequencing 2](#_Toc123667760)

[Supplementary Material 2 – SARS-CoV-2 molecular epidemiology analysis 2](#_Toc123667761)

# Supplementary Material 1 – SARS-CoV-2 genomic sequencing

Samples were sequenced using Oxford Nanopore technology following the ARTIC network V3 protocol (<https://www.protocols.io/view/ncov-2019-sequencing-protocol-v3-locost-bh42j8ye>)

and assembled using the ARTICnetwork assembly pipeline (<https://artic.network/ncov-2019/ncov2019-bioinformatics-sop.html>). All SARS-CoV-2 sequences from this study are available in GenBank (<https://www.ncbi.nlm.nih.gov/genbank/>) under continuous accession numbers OM540759 to OM540803.

Viral RNA was extracted from oro-nasopharyngeal swabs samples using the Maxwell® 16 instrument (Promega, Madison, WI, USA) and directly used for first-strand synthesis using the SuperScript IV Reverse Transcriptase (Life Technologies). PCR-based target enrichment was performed using the ARTIC V3 primer scheme generating 400 bp amplicons in a tiled fashion across the whole genome. For samples with initial cycle threshold (Ct) above 30, containing lower viral templates and typically yielding poorer sequencing results, some modifications were introduced to the protocol at the RNA extraction step (2 aliquots, elution volume of 30µL instead of 60µL), PCR protocol (cDNA volume input increased to 4,4µL instead of 1,25µL) and library preparation (no dilution of amplified cDNA input) steps. A maximum of 24 samples were multiplexed using EXP-NBD104/EXP-NBD114 kits prior to sequencing for at least 40h on portable MinION sequencers using FLO-MIN106 (v9.4.1) flow cells (Oxford Nanopore Technologies, Oxford, UK). To accelerate results reporting, fast live basecalling and demultiplexing were done using MinKNOW (v20.06.4). Reads filtering (400-700 bp) was performed using the ARTIC Network pipeline. Filtered reads were mapped to the Wuhan-Hu-1 reference genome (accession MN908947.3) using minimap2 (V2.17) to generate a consensus sequence with annotated single nucleotide polymorphism (SNP) using a minimal sequencing depth of 20x as a threshold and a 50·1% cutoff for defining SNPs. Unipro Ugene (v36) and Treetime (V0.9.2) were used for SNP distance matrix building, phylogenetic analysis and visualization.

# Supplementary Material 2 – SARS-CoV-2 molecular epidemiology analysis

All included samples were randomly matched with two (community and hospital) contemporary positive samples to generate a viral genome back catalogue and better contextualize phylogenetic analyses findings. All COVID-19 cases were included in the outbreak analyses regardless of sequencing performance. To ensure rapid resulting of actionable information to IPAC teams, available samples were sequenced in multiplexed batches as they became available, and outbreak phylogenetic analyses were iteratively complemented with newly obtained samples.

To confirm genetically related strains, samples were included in the analysis if their coverage at 20x was at least 85%. To refute transmission, samples yielding a sequencing depth of 20x over between 50% and 85% of the genome were included if genomic variability within these appropriately covered genomic loci was above the pre-specified thresholds. Conversely, if suggested to belong to a transmission cluster based on the pre-specified “likely” or “possible” transmission threshold, those isolates were excluded because of potentially unrecognized SNPs within the missing genomic regions. All samples with less than 50% coverage at 20x were excluded.

We did not consider potential within host vial subpopulations when establishing consensus sequences. ^1^

**References**

**1.** James SE, Ngcapu S, Kanzi AM, et al. High Resolution analysis of Transmission Dynamics of Sars-Cov-2 in Two Major Hospital Outbreaks in South Africa Leveraging Intrahost Diversity. *medRxiv* 2020.
